# A comprehensive analysis of the prognostic and immunotherapeutic characteristics of KIFC1 in pan-cancer and its role in the malignant phenotype of pancreatic cancer

**DOI:** 10.18632/aging.205311

**Published:** 2023-12-18

**Authors:** Shihang Zhang, Ouyang Qin, Huanming Xu, Shu Wu, Wei Huang, Hailiang Song

**Affiliations:** 1 Department of General Surgery, Dalang Hospital, Dongguan, Guangdong, PR China; 2 Department of Hepatic-Biliary-Pancreatic Surgery, The Affiliated Dongguan Hospital Southern Medical University (Dongguan People’s Hospital), Dongguan, Guangdong, PR China; 3 Department of Hepatic-Biliary-Pancreatic Surgery, The Affiliated Hospital of Guizhou Medical University, Guiyang, PR China

**Keywords:** KIFC1, pan-cancer, prognosis, immune infiltration, pancreatic cancer

## Abstract

Background: Kinesin family member C1 (KIFC1) is an essential member of the motor protein family, which is critically involved in various cellular events, such as mitosis, meiosis, and macromolecular transport, but also in carcinogenesis, malignant progression, and tumor recurrence.

Methods: The analysis determined the relationship between KIFC1 expression, prognosis significance, immune characteristics landscape, and genetic alterations in pan-cancer with the data extracted from web-based platforms and databases, including but not limited to UCSC, NCBI, GEPIA2, HPA, cBioPortal, SangerBox, UALCAN, GEO and TCGA. Additionally, the expression of KIFC1 in pancreatic cancer tumor tissues and adjacent normal tissues was evaluated through immunohistochemistry. *In vitro* Edu, colony formation, wound healing, and Transwell assay were done to elucidate the biological functions of KIFC1 in pancreatic cancer cells.

Results: The analysis revealed that KIFC1 is upregulated in most cancers, and its increased expression is significantly associated with reduced overall survival and disease-free survival in multiple cancer types. Additionally, strong correlations between KIFC1 expression and tumor immunotherapy were observed across various malignancies. Through univariate and multivariate Cox regression analyses using TCGA data, KIFC1 was identified as an independent predictor of prognosis in pancreatic cancer cases. Furthermore, cellular experiments demonstrated that knockdown of KIFC1 resulted in the suppression of cell proliferation, migration, and invasive ability.

Conclusions: Our study indicated that KIFC1 harbors the potential to be a prognostic and immunotherapeutic biomarker of tumors, and it can have an impact on the metastasis and the cell cycle of pancreatic cancer cells.

## INTRODUCTION

Pancreatic cancer is considered a severe form of gastrointestinal malignancy characterized by an unfavorable prognosis resulting from delayed diagnosis, ineffective treatment options, high recurrence rates, and persistent resistance to chemotherapy [1]. Epidemiologically, pancreatic ductal adenocarcinoma (PDAC), accounting for approximately 95% of pancreatic cancer, represents the seventh-leading cause of cancer-associated death worldwide, and its global burden has more than doubled over the past 25 years. Although genetics and aging are considered irresistible risk factors for this fatal disease, several modifiable risk factors include cigarette smoking, obesity, alcohol intake, long-standing diabetes, and pancreatitis [2]. Pancreatic cancer is endowed with the highest mortality rate owing to its early-stage asymptomatic character, aggressivity, and metastatic capacity, rendering patients to fail to undergo radical surgery. Even if performed with early surgical resection, more than 90% of patients will have a recurrence [3]. The primary strategy for achieving an improved prognosis for patients with PDAC involves the identification of new biomarkers for diagnosis and prognostication purposes, the development of a practical and efficacious predictive system, and the active pursuit of scientifically rigorous clinical interventions.

Appropriate cellular morphology and function are conditional upon the methodical intracellular transport system. Kinesin superfamily proteins (KIFs), also known as molecular motors, have been identified to transport organelles, protein complexes, and mRNAs to specific destinations along microtubules while hydrolyzing ATP for energy [4, 5]. Early in 1992, Originally discovered in a chromosomal segment positioned centromeric to the gene area encoding class II components of the primary human histocompatibility complex [6], the kinesin family member C1 (KIFC1) is a minus-end-directed motor protein, which belongs to the kinesin-14 family. Mounting studies have witnessed that aberrant expression and regulation of kinesin family genes results in the generation and development of various human cancers [7].

KIFC1, functioning as a motor protein, utilizes ATP energy to slide along microtubules. It performs rapid microtubule sliding, thereby facilitating cell division, organelle transport, and cytoplasmic substance localization [8, 9], Furthermore, KIFC1 possesses the capability to crosslink microtubules, effectively impeding the sliding of other motors on them. During mitosis, KIFC1 is involved in centrosome separation and spindle formation. It localizes at the plus-ends of cytoplasmic microtubules and aids in early-stage centrosome detachment, contributing to the assembly and stabilization of the spindle fibers [10, 11]. As a member of KIFs, KIFC1 plays a pivotal role not only in various cellular events but also in carcinogenesis, malignant progression, and tumor recurrence. Recent studies have revealed that KIFC1 could be a potential target for clinical diagnosis and therapeutic alternative for various tumors. Indeed, KIFC1 is observed to be highly expressed and associated with poor prognoses in several solid tumors such as esophageal squamous cell carcinoma [12], non-small cell lung cancer [13], meningioma [14], hepatocellular carcinoma [15], pancreatic ductal adenocarcinoma [16], renal cell carcinoma [17], triple-negative breast cancer [18], serous ovarian adenocarcinoma [19], prostate cancer [20], endometrial cancer [21], bladder cancer [22]. Kwon et al. discovered that the remarkable increase of multipolar anaphases and the selective cell death with centrosome amplification resulting from the depletion of KIFC1 provided a potential new target for effective cancer therapy [23]. Interestingly, KIFC1 still participates in cisplatin resistance in bladder cancer [22], docetaxel resistance in breast cancer [24] and prostate cancer [25], and temozolomide (TMZ) resistance in glioblastoma [26].

Given the pivotal involvement and regulatory functions of KIFC1 in oncogenesis, it is imperative to conduct a comprehensive and extensive pan-cancer analysis to establish the potential role of KIFC1 across various tumor types. In this regard, we conducted a thorough and systematic bioinformatics inquiry to determine the biological functions as well as the prognostic significance of KIFC1 across multiple types of malignancies. Leveraging data sourced from numerous databases, we comprehensively investigated the functional implications of perturbations in KIFC1 expression levels in diverse cancer phenotypes, incorporating crucial clinical parameters such as predictive outcome, genetic modifications, levels of gene expression linked to KIFC1, and immune cell infiltration. Importantly, our bioinformatics results were validated using rigorous *in vitro* experimentation techniques to ensure robustness and fidelity.

## MATERIALS AND METHODS

### Differential expression analysis

We extracted the transcriptome data of cancer samples from The Cancer Genome Atlas (TCGA) and Gene Expression Omnibus (GEO) databases and executed analyses on the disparity of KIFC1 expression between normal and tumorous tissues. Then, to undertake supplementary analyses aimed at elucidating the expression differences between normal and tumor tissues, we employed an integrated approach that combined data from the TCGA database with GTEx data. This analytical framework enabled us to comprehensively investigate gene expression patterns across diverse tissue types using the SangerBox tool (http://sangerbox.com/home.html, accessed on 10 February 2023). To gain further insight into the KIFC1 expression profiles in tumor tissues, we conducted a paired analysis to compare its expression levels with those observed in corresponding adjacent tissues. Furthermore, we conducted a survey on UALCAN (http://ualcan.path.uab.edu/analysis-prot.html, accessed on 12 February 2023) to investigate the potential association between KIFC1 expression levels and tumor staging utilizing the CPTAC dataset to perform visual analysis.

### Survival prognostic analysis

We conducted a robust investigation of overall survival (OS), disease-free survival (DFS), disease-free interval (DFI), and progression-free interval (DFI) among 33 distinct tumor types through the “surv-cutpoint” function of the “survminer” R package. Patients were categorized into high and low groups by employing a “median” group cutoff, enabling the generation of Kaplan-Meier survival curves and maps. Additionally, we calculated log-rank *p*-values and hazard ratios (HRs) to systematically evaluate the prognostic relevance of KIFC1 expression levels across diverse tumor types.

### Gene set enrichment analysis

The hallmark gene set “h.all.v7.4.symbols.gmt,” which comprises 50 gene sets, was obtained from the Molecular Signatures Database (MSigDB) website (https://www.gsea-msigdb.org/gsea/index.jsp). This file was utilized to perform Gene Set Enrichment Analysis (GSEA) on the differentially expressed genes (DEGs) between the low and high-KIFC1 expression cancer groups. The GSEA was conducted using the R package “clusterProfiler,” and the normalized enrichment score (NES) and false discovery rate (FDR) were calculated for each biological process within each cancer type. The results of the GSEA analysis were then summarized in a bubble plot using the R package “ggplot2.”

### Immune cell infiltration analysis

The immune cell infiltration levels of TCGA cancers were obtained from the TIMER2 database (http://timer.cistrome.org/). Spearman correlation analysis was conducted to investigate the relationships between KIFC1 mRNA expression and 21 immune cell subsets across different cancer types. These immune cell subsets include CD4^+^ T cells, cancer-associated fibroblasts (CAF), lymphoid progenitors, myeloid progenitors, monocyte progenitors, endothelial cells (Endo), eosinophils (Eos), hematopoietic stem cells (HSC), T-cell follicular helper cells, γδ T cells, NK T cells, regulatory T cells (Tregs), B cells, neutrophils, monocytes, macrophages, dendritic cells, NK cells, mast cells, and CD8^+^ T cells. The analysis aimed to explore potential associations between KIFC1 mRNA expression and immune cell infiltration levels in a pan-cancer context.

Tumor Immune System Interaction Database (TISIDB) is a public, online database that provides information on the expression profiles, precise regulatory mechanisms, and clinical relevance of a wide range of immune genes in tumor and normal tissues. Using the TISIDB (http://cis.hku.hk/TISIDB/) database, an analysis was performed to explore the correlation between KIFC1 and the tumor immune microenvironment (TIME), which encompasses tumor-infiltrating lymphocytes, immunocyte co-inhibitors, and co-stimulators.

### Immunotherapy prediction analysis

Spearman correlation analysis was applied to evaluate the statistical correlations between KIFC1 and established immunotherapy biomarkers, such as tumor mutation burden (TMB), microsatellite instability (MSI), and other well-known immune checkpoint genes, across various cancer types in a pan-cancer analysis. Additionally, we acquired immune checkpoint blockade (ICB) therapy cohorts, specifically the IMvigor210 cohort consisting of 288 urological cancer patients treated with atezolizumab (anti-PDL1), to validate the predictive capability of KIFC1 in determining the response to immunotherapy.

### Protein network construction and gene enrichment analysis

The functionally identical genes based on genomic and proteomic data can be found through the GeneMANIA database [27]. To gain insight into the potential functions of KIFC1, we utilized the GeneMANIA database to predict genes with similar functionalities. Additionally, Gene Ontology (GO) and Kyoto Encyclopedia of Genes and Genomes (KEGG) analyses were performed on molecular entities that demonstrated potential involvement with KIFC1.

### Correlation analysis of KIFC1 expression with clinical characteristics

We conducted a comprehensive analysis to investigate the link between the expression of KIFC1 and various clinical stages, including pathologic and tumor stages, lymph node stage, metastasis stage, age, et al. Next, the univariate and multivariate Cox regression and Receiver Operating Characteristic Curve (ROC) analysis were conducted to evaluate the prognostic value of KIFC1 in pancreatic cancer patients. To enhance the utility of KIFC1 as a prognostic biomarker in pancreatic cancer, we developed a nomogram based on both KIFC1 expression levels and pathologic stage, and the calibration curve evaluated its prediction accuracy of the nomography.

### Cell culture

The Human Pancreatic Ductal Epithelial (HPDE), PDAC cell lines PANC-1, SW1990, and MIA PaCa-2 for this experiment were purchased from the American Type Culture Collection (ATCC, Manassas, VA, USA). Those kinds of cells were cultured at 37, 5% CO2 in DMEM (added 10% FBS and 1% penicillin/streptomycin). The small interfering RNA (siRNA) of the KIFC1 gene was purchased from DesignGene company (http://www.designgene.com.cn/) to knockdown the expression of KIFC1 in PANC-1 and SW1990 cells by being transfected with lipo3000, and the sequence is as follows:

**Table:** 

siRNA-KIFC1	5′-CCUGGAGCCUGAGAAGAAATT-3′ sense
	5′-UUUCUUCUCAGGCUCCAGGTT-3′ antisense
Negative control (NC)	5′-UUCUCCGAACGUGUCACGUTT-3′ sense
	5′-ACGUGACACGUUCGGAGAATT-3′ antisense

### Western blot analysis

Protein concentrations were tested via the BCA way. Western blotting was applied for the detection of the protein level of E-cadherin, N-cadherin, Vimentin, BAX, BLC-2, and the KIFC1 after knocking down KIFC1 in PANC-1 and SW1990 cells.

### Edu assay

The 4 × 10^5^ cells of each group per well in 6-well plates were maintained with 20μM for two hours. Next, the samples were treated with 4% paraformaldehyde for a duration of 20 minutes before being thoroughly rinsed thrice in 0.5% Triton X-100 solution. Subsequently, the Click reaction was finished according to BeyoClick™ EdU Cell Proliferation Kit with Alexa Fluor 555 (https://www.beyotime.com/product/C0075L.html). Ultimately, the resulting image was captured utilizing a microscope.

### Colony formation assay

Each group supplied 1,000 cells per well and seeded in 6-well plates for two weeks of incubation in a 37°C, 5% CO_2_ incubator. After two weeks, the cells were fixed with 4% paraformaldehyde for 20 minutes and stained with crystal violet for 30 minutes. Three multiple wells were designed for each group.

### Wound healing assay

After the siRNA-transfected cells in the 6-well plate grew to almost 100%, the cells were scratched in the plate with the 200 μL pipetting head and replaced with the serum-free medium to avoid cell growth effects. Subsequently, the image was captured with a microscope at 0 h, 24 h, and 48 h after scratching.

### Transwell migration and invasion assay

According to matrix glue: Serum-free medium = 1:8, the upper chamber of Transwell was covered with a stratum of adhesive Matrigel matrix (Corning Company, Corning, NY, USA), and the lower chamber was covered with 700 μL complete medium. Then, 5 × 10^4^ cells of each group in 200 μL serum-free medium were seeded in the upper chamber and then cultured in an incubator (37°C, 5% CO_2_) for 24 h. Next, the cells underwent fixation with 4% paraformaldehyde for a period of 20 minutes, after which they were subjected to staining with crystal violet for 30 minutes. Sterile cotton was used to wipe off the cells remaining in the upper chamber slightly. The Transwells were dried at room temperature and photographed under a microscope. The distinguishing factor between the Transwell invasive assay and migration assay is exclusively attributed to the presence or absence of a Matrigel matrix adhesive layer.

### Statistical analysis

The results of the statistical analyses were visualized using GraphPad Prism 9.0 software and R software (version 4.3.0). Spearman analysis was used to evaluate correlation coefficients. For normally distributed continuous variables, independent sample tests were employed, while Mann-Whitney *U* tests were used for non-normally distributed continuous variables. Data comparison among the three groups was carried out using one-way ANOVA. Survival analysis was conducted using the Kaplan-Meier method with a two-sided log-rank test. A *p*-value less than 0.05 was considered statistically significant (^*^p < 0.05; ^**^p < 0.01; ^***^p < 0.001; ^****^p < 0.0001).

### Data availability statement

This article features the original research contributions presented by our study. For any additional inquiries, interested parties may contact the corresponding author directly.

## RESULTS

### The KIFC1 expression in pan-cancer

**Figure 1:**
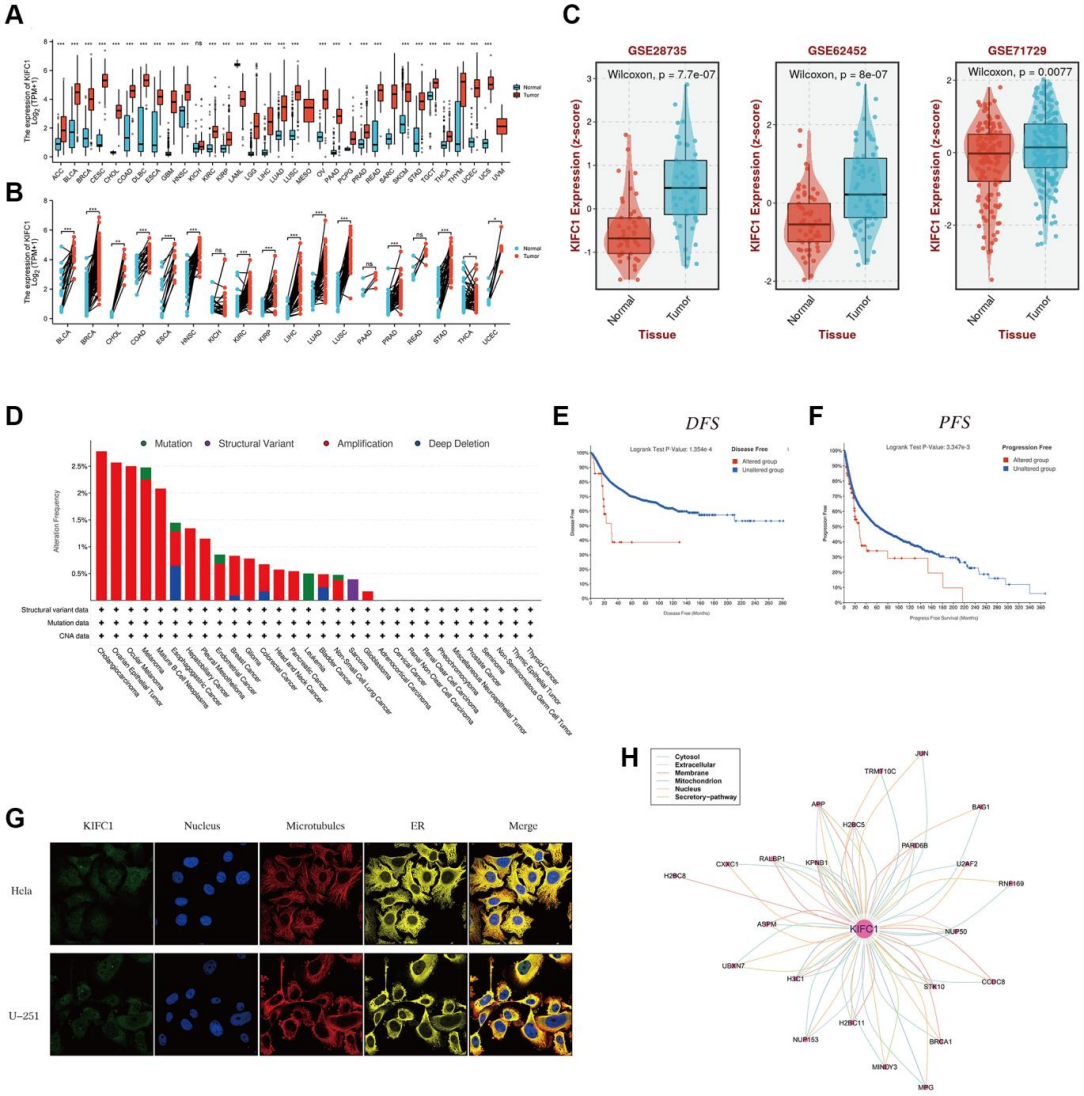
**The expression level of KIFC1 in pan-cancer.** (**A**) KIFC1 expression levels in different cancers with TCGA combined with GTEx database. (**B**) A paired comparison analysis was conducted using the Mann-Whitney *U* test to analyze the expression differences of KIFC1 in the tumor and corresponding adjacent tissues. (**C**) KIFC1 expression levels in GSE26735, GSE62452 and GSE71729. (**D**) Genetic alteration landscape (Mutation, Structural Variant, Amplification, and Deep Deletion) of KIFC1 in 30 different tumors based on the TCGA database through the cBioPortal tool. (**E**) Prognosis of DFS between KIFC1 altered and unaltered groups. (**F**) Prognosis of PFS between KIFC1 altered and unaltered groups. (**G**) The immunofluorescence images of the KIFC1 protein, nucleus, endoplasmic reticulum (ER), microtubules, and the merged images in Hela and U251 cell lines. (**H**) The protein-protein interaction (PPI) network presents the proteins interacting with KIFC1 ns *p* ≥ 0.05; ^*^p < 0.05; ^**^p < 0.01; ^***^p < 0.001. ^****^p < 0.0001.

The expression levels of KIFC1 in various cancers were assessed compared to normal tissues using the TCGA and GTEx databases. The analysis revealed that high expression of KIFC1 was observed in a majority of TCGA cancers, including ACC, BLCA, BRCA, CESC, CHOL, COAD, DLBC, ESCA, GBM, HNSC, KIRC, KIRP, LAML, LGG, LIHC, LUAD, LUSC, OV, PAAD, PCPG, PRAD, SKCM, STAD, TGCT, THCA, THYM, UCEC, and UCS. Conversely, low expression of KIFC1 was specifically detected in LAML (Figure 1A). Furthermore, paired comparison analysis was performed, and the results revealed the relative overexpression of KIFC1 in BLCA, BRCA, CHOL, COAD, ESCA, HNSC, KIRC, KIRP, LIHC, LUAD, LUSC, PRAD, STAD, THCA, UCEC in tumor tissues (Figure 1B). Compared to normal tissues, the mRNA expression of KIFC1 was upregulated in PAAD according to external datasets such as GSE26735, GSE62452, and GSE71729 (Figure 1C).

Genetic alterations, incorporating the mutations, deletions, or amplification phenomena in oncogenes or tumor suppressor genes, may lead to phenotypical changes that have the potential to initiate a tumor and promote its progression. Hence, we first analyzed the landscape of genetic alterations such as mutation, structural variant, amplification, and deep deletion in the KIFC1 gene using the TCGA cancer datasets through the cBioPortal tool. The KIFC1 genetic alteration profiles exhibit those amplifications (>2.5%) in cholangiocarcinoma and ovarian epithelial tumors ranked among the best (Figure 1D). To analyze the impacts of KIFC1 alterations on the prognosis of patients, we found that patients in the KIFC1 altered group have shorter DFS and PFS compared to those in the KIFC1 unaltered group (Figure 1E, 1F). According to the provided information, immunofluorescence (IF) images demonstrated that the KIFC1 protein exhibited predominant localization in the centrosome of Hela and U251 tumor cell lines (Figure 1G). Additionally, a protein-protein interaction (PPI) network was constructed using interaction data obtained from the ComPPI website, which revealed that proteins closely associated with KIFC1 were distributed in various subcellular compartments, including cytosol, mitochondria, nucleus, extracellular space, secretory pathway, and membrane (Figure 1H).

### Survival analysis of KIFC1 in the pan-cancer

**Figure 2:**
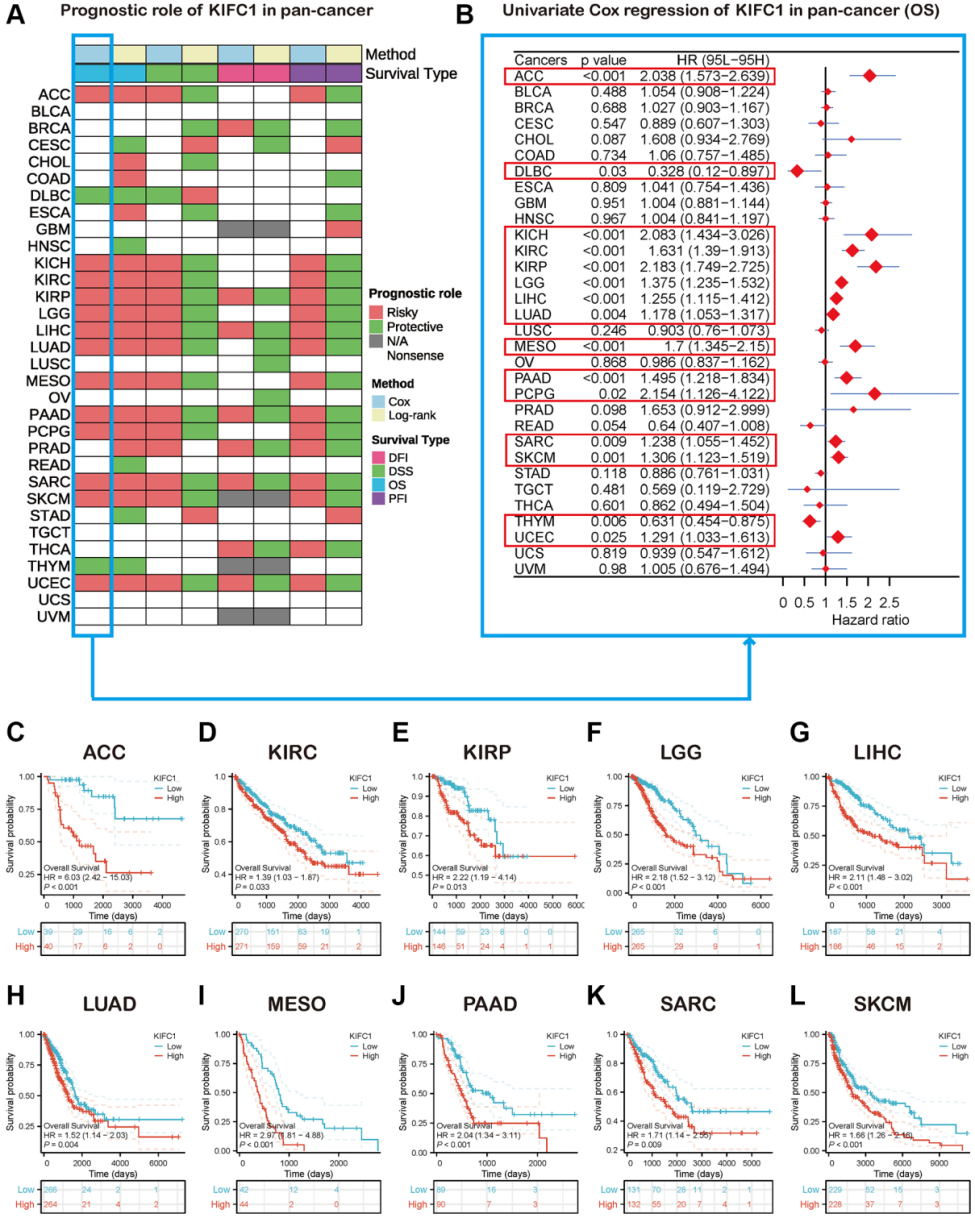
**Survival analysis of KIFC1 in the pan-cancer.** (**A**) Summary of the correlation between expression of KIFC1 with overall survival (OS), disease-specific survival (DSS), disease-free interval (DFI), and progression-free interval (PFI) based on the univariate Cox regression and Kaplan-Meier models. (**B**) The forest plot exhibited the prognostic role of KIFC1 in cancers by the univariate Cox regression method. (**C**–**L**) Kaplan-Meier overall survival curves of KIFC1 in ACC, KIRC, KIRP, LGG, LIHC, LUAD, MESO, PAAD, SARC, and SKCM.

The heatmap analysis of KIFC1 in pan-cancer demonstrated a strong association between KIFC1 and the prognosis of most cancers (Figure 2A). Overall survival (OS) analysis revealed that KIFC1 acts as a detrimental factor for patients with ACC, KICH, KIRC, KIRP, LGG, LIHC, LUAD, MESO, PAAD, PCPG, SARC, SKCM, and UCES, while it serves as a protective factor for patients with DLBC and THYM. To further understand how KIFC1 affects patient prognosis, univariate Cox regression analysis was performed across 32 TCGA cancer types. The results depicted in the forest plot indicated that downregulation of KIFC1 expression significantly correlated with extended OS time in ACC (HR = 2.038 (95% CI, 1.573 to 2.639), *p* < 0.001), DLBC (HR = 0.328 (95% CI, 0.12 to 0.897), *p* = 0.03), KICH (HR = 2.083 (95% CI, 1.434 to 3.026), *p* < 0.001), KIRC (HR = 1.631 (95% CI, 1.39 to 1.913), *p* < 0.001), KIRP (HR = 2.183 (95% CI, 1.749 to 2.725), *p* < 0.001), LGG (HR = 1.375 (95% CI, 1.235 to 1.532), *p* < 0.001), LIHC (HR = 1.255 (95% CI, 1.115 to 1.412), *p* < 0.001), LUAD (HR = 1.178 (95% CI, 1.053 to 1.317), *p* = 0.004), MESO (HR = 1.7 (95% CI, 1.345 to 2.15), *p* < 0.001), PAAD (HR = 1.495 (95% CI, 1.218 to 1.834), *p* < 0.001), PCPG (HR = 2.154 (95% CI, 1.126 to 4.122), *p* = 0.02), SARC (HR = 1.238 (95% CI, 1.055 to 1.452), *p* = 0.009), SKCM (HR = 1.306 (95% CI, 1.123 to 1.519), *p* = 0.001), THYM (HR = 0.631 (95% CI, 0.454 to 0.875), *p* = 0.006), and UCES (HR = 1.291 (95% CI, 1.033 to 1.612), *p* = 0.025) (Figure 2B). Moreover, Kaplan-Meier survival analysis confirmed that elevated KIFC1 expression in ACC, KIRC, KIRP, LGG, LIHC, LUAD, MESO, PAAD, SARC, and SKCM was associated with poor OS prognosis (Figure 2C–2L).

### GSEA of KIFC1 in pan-cancer

**Figure 3:**
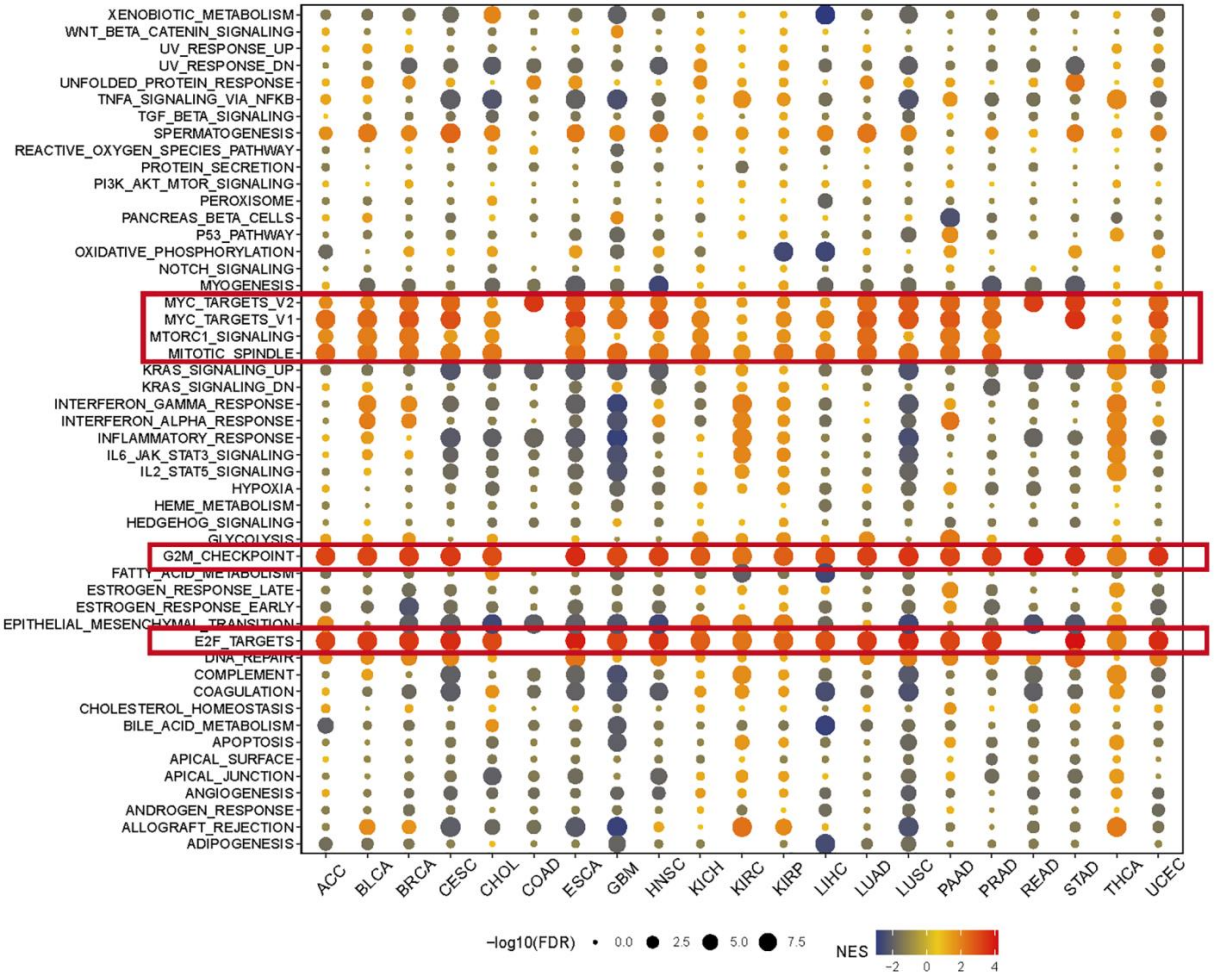
**The hallmarks gene set enrichment analysis of KIFC1 in pan-cancer.** The size of each circle represents the false discovery rate (FDR) value of the enriched term in each specific cancer, while the color indicates the normalized enrichment score (NES) of the corresponding term.

GSEA was performed using the differentially expressed genes (DEGs) between low- and high-KIFC1 subgroups in each cancer to identify the cancer hallmarks associated with KIFC1. The analysis revealed a significant correlation between KIFC1 expression and MYC targets, E2F targets, Mitotic spindle, G2M checkpoint, and MTORC1 signaling in the majority of cancer types (Figure 3). These findings suggest a potential connection between KIFC1 expression cell cycle regulation and cell proliferation. In summary, these results indicate that higher expression of KIFC1 is linked to growth regulation in various cancers and offer insights for further investigation into the functions and roles of KIFC1 in cancer initiation and progression.

### Expression of KIFC1 and analysis of tumor microenvironment

**Figure 4:**
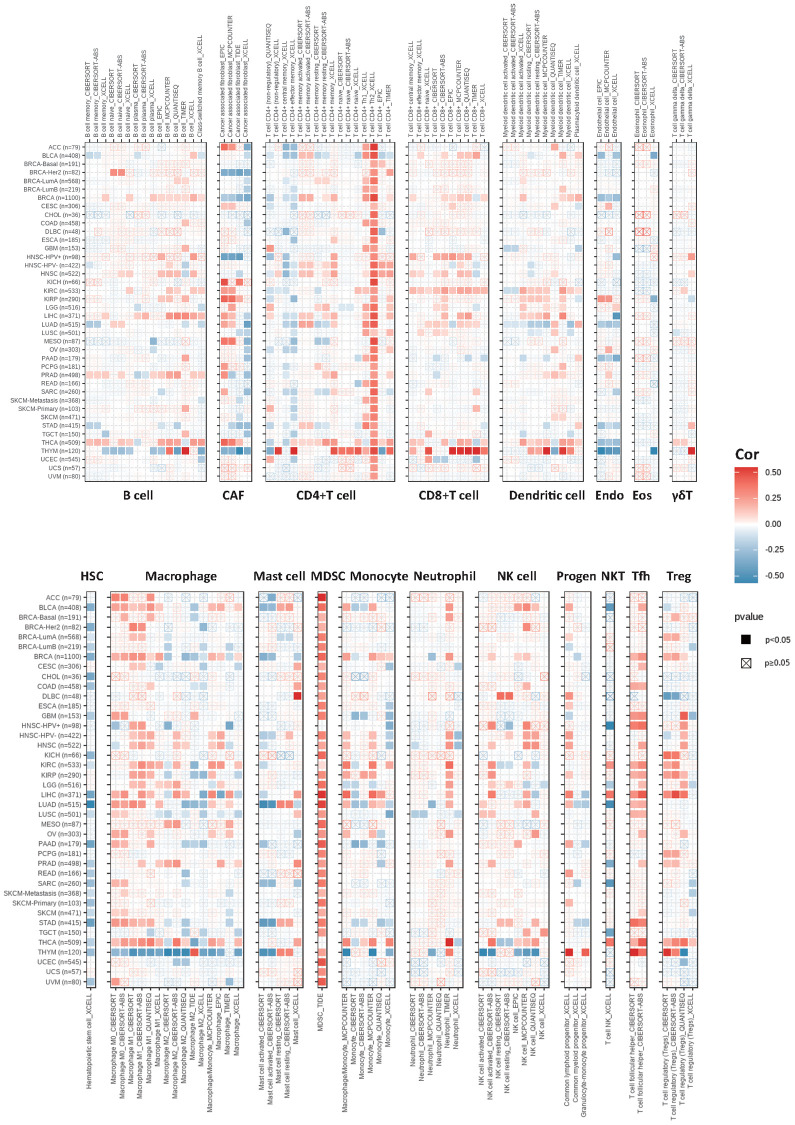
**The correlations of KIFC1 expression and the infiltration levels of B cells, CAFs, CD4+ T cells, CD8+ T cells, dendritic cells, Endo, Eos, γδT cells, HSCs, macrophages, mast cells, myeloid-derived suppressor cells (MDSCs), monocytes, neutrophils, lymphoid progenitor cells, myeloid progenitor cells, monocyte progenitor cells, Tfh, NK T cells, and Tregs.** Positive correlation in red and negative correlation in blue.

The tumor microenvironment encompasses a milieu of immune cells, mesenchymal components, and an internal and external molecular composition that exerts a critical influence on the initiation, progression, metastasis, and therapeutic response of tumors [28]. Prior investigations have revealed the prognostic significance and degree of expression of KIFC1 in a range of cancer types; however, the influence of abnormal expression of KIFC1 on immune cell infiltration is still ill-defined. To explore the relationship between KIFC1 and cancer immunity, we investigated the association between KIFC1 expression and immune cell infiltrations. To accomplish this objective, Spearman correlation analyses were performed using pan-cancer immune cell infiltration data obtained from the TIMER2 database. The results demonstrated correlations between KIFC1 expression and the infiltration levels of various immune cell types, including B cells, cancer-associated fibroblasts (CAFs), CD4^+^ T cells, CD8^+^ T cells, dendritic cells, endothelial cells (Endo), eosinophils (Eos), γδT cells, hematopoietic stem cells (HSCs), macrophages, mast cells, myeloid-derived suppressor cells (MDSCs), monocytes, neutrophils, lymphoid progenitor cells, myeloid progenitor cells, monocyte progenitor cells, T follicular helper cells (Tfh), NK T cells, and regulatory T cells (Tregs) in pan-cancer. (Figure 4). The findings revealed a positive correlation between KIFC1 expression and the infiltration levels of CD4+ T cells, CD8+ T cells, myeloid-derived suppressor cells (MDSCs), T follicular helper cells (Tfh), and macrophages in the majority of TCGA cancers. Conversely, KIFC1 showed a negative association with the infiltration levels of NK T cells and HSCs, particularly in BLCA, LUAD, and SARC among the TCGA cancers. These results suggest that KIFC1 may play a role in the development, prognosis, and treatment of cancers by interacting with immune cells.

### The role of KIFC1 in cancer immunotherapy

**Figure 5:**
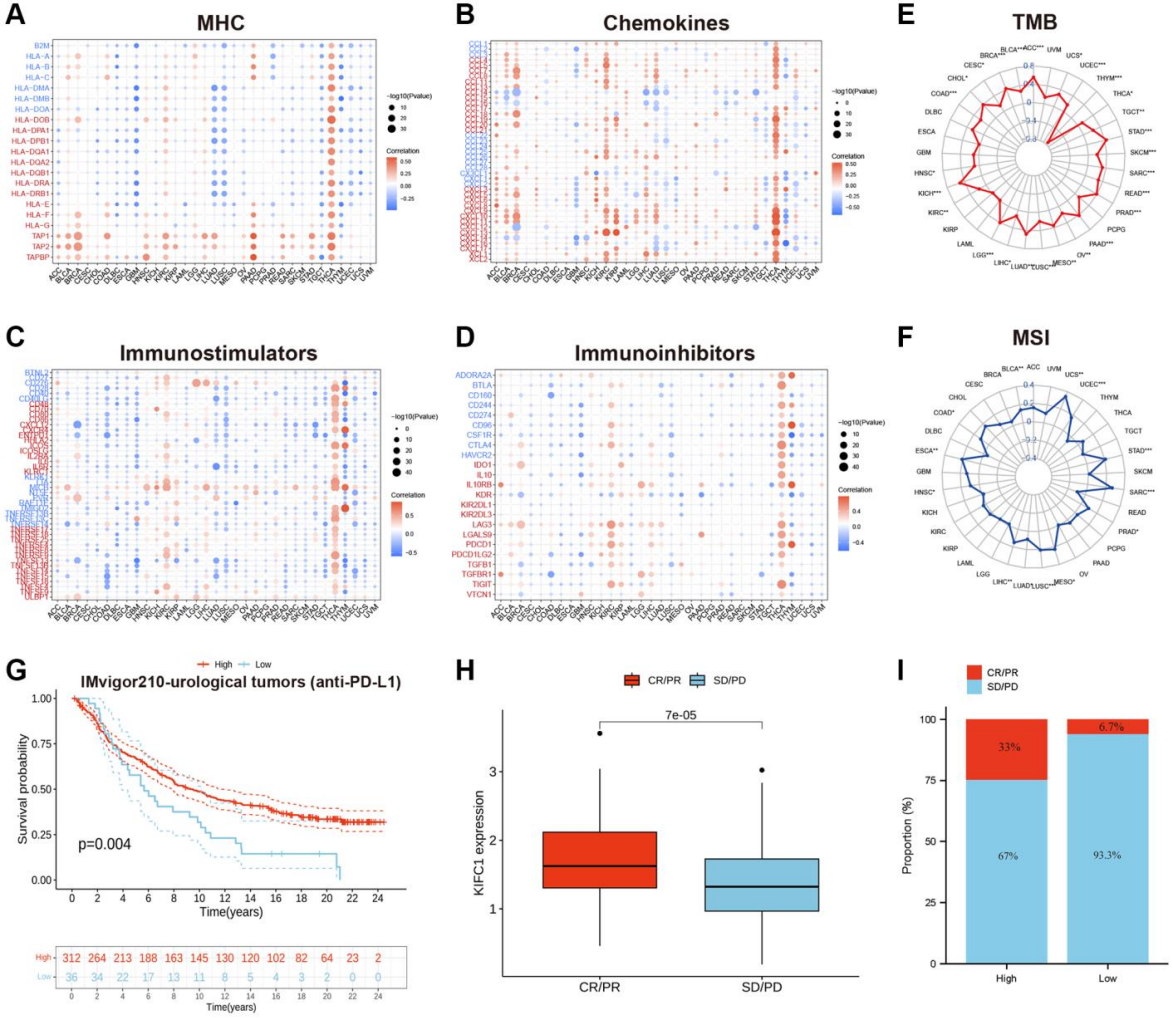
(**A**) The correlations between the KIFC1 expressions and MHC molecule in pan-cancer. (**B**) The correlations between the KIFC1 expressions and chemokines in pan-cancer. (**C**) The correlations between the KIFC1 expressions and immunostimulators in pan-cancer. (**D**) The correlations between the KIFC1 expressions and immunoinhibitors in pan-cancer. Red represents a positive correlation, and blue represents a negative correlation. (**E**) Association between KIFC1 expression and TMB. (**F**) Association between KIFC1 expression and MSI. (**G**) Kaplan-Meier curves for low- and high-KIFC1 patient groups in IMvigor210 cohort. (**H**) The differential expression of KIFC1 in the CR/PR group and SD/PD group. (**I**) The fraction of urological tumors patients with response to anti-PD-1 therapy in low- and high-KIFC1 subgroups of IMvigor210 cohort.

To investigate the role of KIFC1 in modulating the cancer immune microenvironment, we conducted correlation analyses between three kinds of immunomodulators as well as chemokines and expression of KIFC1 at the pan-cancer level. The results demonstrated that the expression of KIFC1 was significantly positively related to MHC molecules, chemokines, immunostimulators, and immunoinhibitors in several types of cancer, especially in THCA (Figure 5A–5D). Additionally, tumor mutation burden (TMB) and microsatellite instability (MSI) are developing as immunotherapy biomarkers [29, 30]. Hence, we observed the link between TMB/MSI and KIFC1 expression in pan-cancer, and we found that KIFC1 was positively associated with TMB in most cancers while negatively linked with TMB in THYM. In addition, KIFC1 was positively associated with MSI in BLCA, COAD, HNS, LIHC, LUAD, LUSC, and MESO. PRAD, SARC, STAD, UCEC, and UCS, but the correlation is not strong (Figure 5E, 5F).

Immune checkpoint inhibitors (ICIs), including anti-PD-L1 antibodies, have revolutionized cancer immunotherapy by significantly improving treatment outcomes. In the IMvigor210 cohort, a study focused on urological tumors, the relationship between KIFC1 and response to anti-PD-L1 therapy was investigated. The findings demonstrated that patients with high KIFC1 expression had better survival rates and longer survival times compared to those with low expression (Figure 5G). Notably, the response rate to anti-PD-L1 therapy was 33% among patients with high KIFC1 expression, which was significantly higher than the response rate of 6.7% observed in patients with low KIFC1 expression (Figure 5H, 5I). These results highlight the potential of KIFC1 as a predictive biomarker for identifying patients who are more likely to respond positively to anti-PD-L1 therapy in urological tumors.

### Molecular interaction network and enrichment analysis

**Figure 6:**
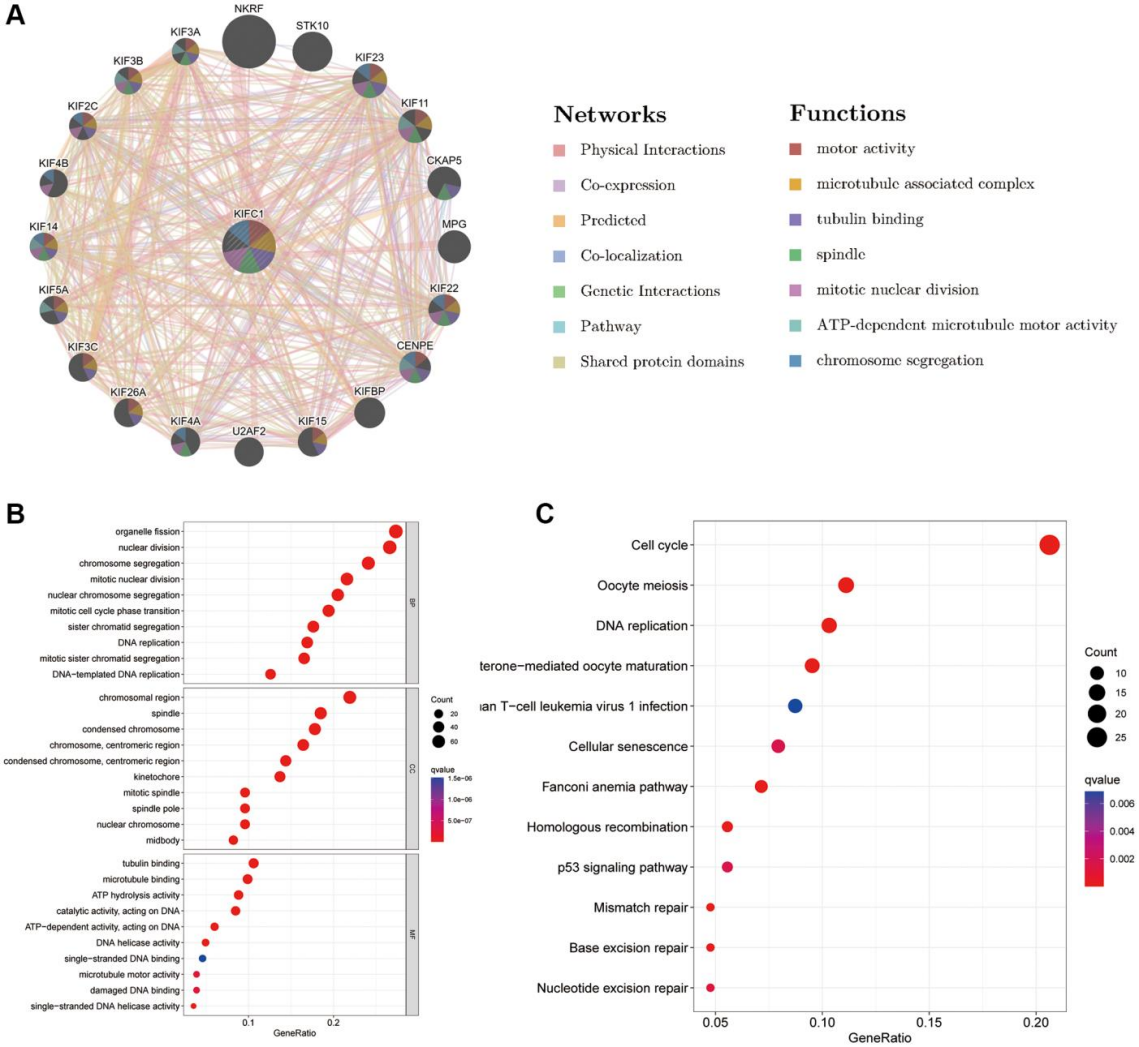
Predicted function of KIFC1 (**A**) The potential interaction molecular network of KIFC1 via GeneMANIA; (**B**, **C**) GO and KEGG functional enrichment analysis of KIFC1.

**Figure 7:**
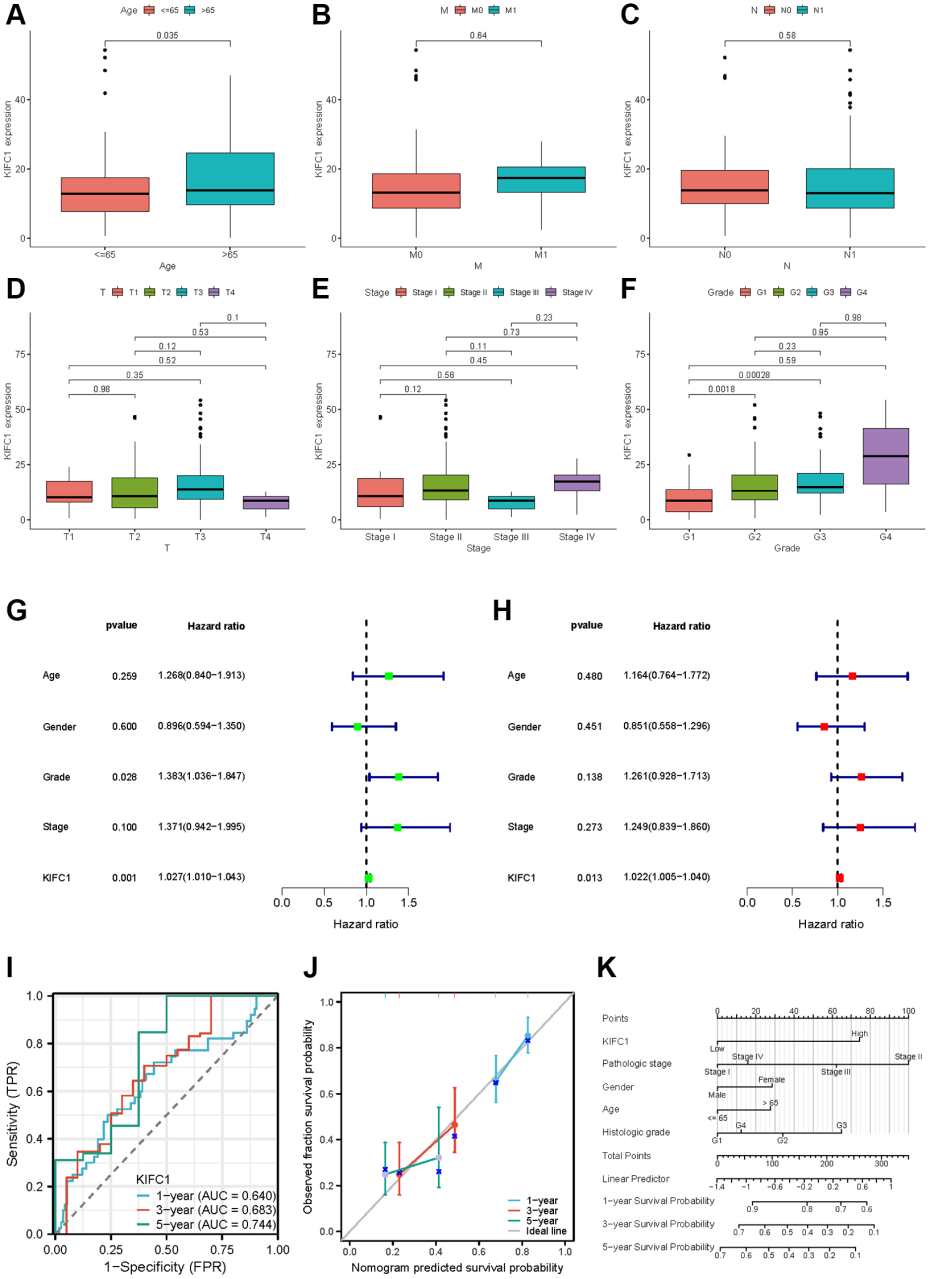
**Clinical characteristics of KIFC1 in pancreatic adenocarcinoma.** Variation analysis of KIFC1 expression in different Ages (**A**), M stage (**B**), N stage (**C**), T stage (**D**), pathological stages (**E**), and histologic grade (**F**). (**G**, **H**) The prognostic significance of KIFC1 in pancreatic cancer was analyzed by univariate and multivariate Cox analysis. (**I**) Prognostic evaluation efficacy of KIFC1 in pancreatic cancer by ROC. (**J**) Calibration curve for evaluating the accuracy. (**K**) A nomogram based on KIFC1 expression and Pathological stage, Gender, Age, and Histologic grade. Ns: *p* ≥0.05; ^*^p < 0.05; ^**^p < 0.01; ^***^p < 0.001. ^****^p < 0.0001.

The deep understanding of the molecules interacting with KIFC1 and the possible role of KIFC1 were studied by GeneMANIA. The results of the network plot from GeneMANIA in Figure 6A revealed that the primary function of KIFC1 included motor activity, microtubule-associated complex, and tubulin binding. KIFC1 might interact with NKRF, STK10, MPG, CENPE, and its family members such as KIF3B, KIF2C, and KIF3C et al. The KEGG and GO enrichment analyses were performed to identify the potential role of KIFC1 further. Biological Processes enrichment analysis showed that these molecules mostly participated in organelle fission, chromosome segregation, nuclear division, and DNA replication (Figure 6B). Cellular Component enrichment analysis displayed that these molecules were mainly linked with the chromosomal region, spindle, and kinetochore (Figure 7B). Molecular Function enrichment analysis revealed that these molecules were prevailingly involved in tubulin binding and ATP hydrolysis activity (Figure 6B). According to the KEGG analysis, it was determined that the aforementioned molecules were largely involved in facilitating cell cycle progression and DNA replication. (Figure 6C).

### Clinical correlation analysis of KIFC1 in pancreatic adenocarcinoma

Our previous study results demonstrate that KIFC1 may be a critical factor in the initiation and development of pancreatic cancer. The results from the analysis of the association between KIFC1 expression and clinical characteristics in pancreatic cancer showed that KIFC1 is relatively highly expressed in the higher age over 65 (Figure 7A) and histologic grade (Figure 7F). There was little difference between the KIFC1 expression and the T, M, N stage (Figure 7B–7D) and the pathologic stage (Figure 7E). The presented data imply a possible association between KIFC1 expression and the malignant pathogenesis of pancreatic cancer. To ascertain the prognostic value of KIFC1 in pancreatic adenocarcinoma (PAAD), univariate and multivariate Cox regression analyses were performed utilizing clinical information, which demonstrated that KIFC1 is an independent prognostic factor in PAAD (Figure 7G, 7H). Additionally, receiver operating characteristic (ROC) analysis was conducted to evaluate the efficacy of KIFC1 as a predictor of overall survival rate in PAAD. KIFC1 was observed as a qualified prognostic evaluation performance in PAAD, with an AUC of 0.640 in 1 year, 0.683 in 3 years, and 0.744 in 5 years (Figure 7I). In addition, we constructed a calibration curve to evaluate the model’s accuracy for assessing prognosis for patients with pancreatic cancer after 1, 3, and 5 years (Figure 7J). A nomogram was constructed based on its expression levels and pathological staging to improve the clinical applicability of KIFC1 in diagnostic assessments. The objective was to provide a quantitative tool for predicting the probability of adverse outcomes with greater accuracy than either of these factors alone (Figure 7K).

### The effects of KIFC1 on malignant phenotypes of pancreatic cancer cells

**Figure 8:**
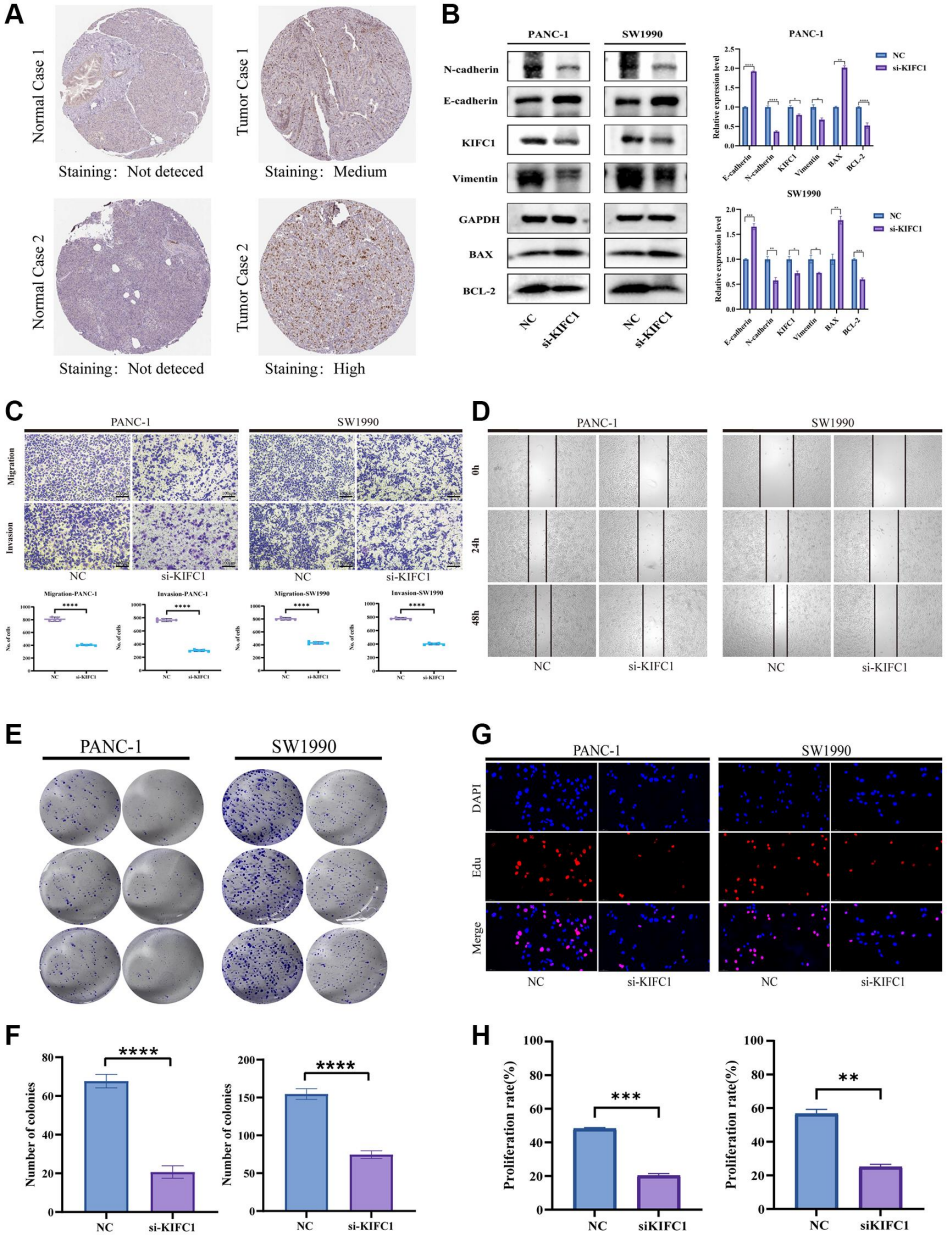
**Effects of KIFC1 on malignant phenotypes of PANC-1 and SW1990 cells.** (**A**) Immunohistochemistry of KIFC1 in pancreatic cancer tumor tissues and corresponding adjacent normal tissues. (**B**) Western blot analysis of KIFC1 knockdown. (**C**) Transwell for testing migration and invasion ability. (**D**) Scratch assay for determining cell migration. (**E**, **F**) Proliferation attenuation of PANC-1 and SW1990 cells was detected by colony formation assay. (**G**, **H**) Proliferation attenuation of PANC-1 and SW1990 cells was detected by Edu assay. Ns: *p* ≥ 0.05; ^*^p < 0.05; ^**^p < 0.01; ^***^p < 0.001. ^****^p < 0.0001.

Based on the previous findings of over-expression of KIFC1 correlated with poor survival expectations in patients with pancreatic cancer, we deeply mined the role of KIFC1 in the carcinogenesis of pancreatic cancer adenocarcinoma. Firstly, drawing upon the immunohistochemical (IHC) data obtained from the HPA database, our preliminary assessment focused on identifying differences in KIFC1 expression between normal pancreatic tissue and pancreatic cancer tissue; our findings suggest that KIFC1 is overexpressed in pancreatic cancer samples as compared to normal pancreatic tissue, wherein no detectable expression was observed (Figure 8A). We investigated the differential expression of KIFC1 in pancreatic cancer cell lines and HPDE by employing Western blot analysis. Our results revealed a significant upregulation of KIFC1 expression in pancreatic cancer cells, whereas its expression was not detected in HPDE cells (Supplementary Figure 1). Afterward, the small interfering RNA to knockdown KIFC1 was constructed and transfected into PANC-1 and SW1990 cells. Subsequently, we set the si-RNA transfected group as si-KIFC1 and the negative vector group as NC in each kind of cell. Western blot analysis figured out the decline of protein level of KIFC1, N-cadherin, vimentin, and Bcl-2, whereas the increase of E-cadherin, BAX at si-KIFC1 group in PANC-1 and SW1990 cells (Figure 8B). Additionally, the Transwell migration and invasion experiments and scratch test were performed to verify the inhibition of migration and invasion ability in pancreatic cells after the ablation of KIFC1 (Figure 8C, 8D). Furthermore, the proliferation ability of si-KIFC1 and NC groups was tested by Edu assay and colony formation assay in PANC-1 and SW1990 cells. The results of the conducted assays demonstrated a noteworthy reduction in the proliferation rate in the si-KIFC1 group relative to the NC group (Figure 8E–8H).

## DISCUSSION

In the present study, we conducted an extensive bioinformatics analysis to investigate the functional significance of KIFC1 across multiple malignancies. The results revealed significant upregulation of KIFC1 in various tumor types, including BLCA, LUAD, PAAD, STAD, OV, and others, as determined through paired and unpaired comparisons using data from TCGA and GTEx cohorts. Additionally, Kaplan-Meier survival analysis demonstrated that patients with elevated KIFC1 expression in ACC, KIRC, KIRP, LGG, MESO, PAAD, and SARC had significantly shorter OS and DFS. These findings suggest that KIFC1 plays a critical role in the pathogenesis and prognostication of malignancies. Through physical interaction analysis performed using the GeneMANIA online platform, significant correlations were observed between KIFC1 and NKRF, STK10, and U2AF2. Furthermore, the KEGG pathway and GO enrichment analyses indicated that KIFC1 may participate in carcinogenesis by influencing key biological processes such as “cell cycle” and “DNA replication” in cancer cells. Immune landscape analysis revealed a positive association between KIFC1 expression levels and the infiltration degree of B cells, neutrophils, and Tregs while showing a negative correlation with the infiltration level of macrophages, CD8^+^ T cells, dendritic cells, and fibroblasts in PAAD.

This study also emphasized the potential of KIFC1 expression as a predictive factor for patient outcomes in pancreatic cancer. Further investigations were carried out to explore the correlation between KIFC1 expression and clinicopathological staging. Moreover, a nomogram was developed to facilitate prognostic evaluation of PAAD patients based on KIFC1 expression levels. *In vitro* experiments utilizing PANC-1 and SW1990 cells demonstrated that knockdown of KIFC1 expression significantly attenuated proliferation, invasion, and migration abilities. Additionally, KIFC1 depletion led to alterations in the expression of Vimentin, N-cadherin, Bcl-2, E-cadherin, and BAX, suggesting that KIFC1 may contribute to carcinogenesis and progression by influencing apoptosis in pancreatic cancer cells.

The tumor immune microenvironment is an integral part of cancer, including tumor cells, immune cells, and cytokines, which significantly function in tumor cell elimination and immune escap [31]. The initial infiltration of immune cells, including macrophages, lymphocytes, natural killer (NK) cells, and dendritic cells (DCs), is an essential factor for the effective control of tumor development [32]. Despite the implementation and clinical use of checkpoint immunotherapies, there exist multiple limitations associated with their current application, including non-responsive patient populations, development of resistance mechanisms, and possible exacerbation of tumor progression [33], blocking the immune checkpoint is still the most promising approach to dampen the malignancies. Lymphocyte activation gene-3 (LAG3) (CD233) is the third inhibitory receptor (IR), whose upregulation is essential to balance co-stimulatory receptor activity and limit T cell activation [34]. CD276 plays an essential role in the inhibition of the T-cell function and is highly expressed among Human solid cancers [35]. Monoclonal antibody-based drugs targeting CTLA4 have been identified as a therapeutic approach to enhance the immune system’s anti-cancer activity [36]. In this study, the expression of KIFC1 was positive with immune checkpoints like CTLA4, CD276, and LAG3 in most tumor types. We preliminarily hypothesize that abnormal expression of KIFC1 regulates the tumor immune microenvironment to generate a poor prognosis.

In animal cells, the centrosome is a vital organelle that performs various essential functions in cellular division. These functions include microtubule nucleation, proper spindle pole formation and orientation, accurate alignment of microtubules, and equitable segregation of chromosomes during mitosis [37]. Reportedly, KIFC1, a Kinesin-14 motor protein, has been implicated in the process of centrosome clustering, whereby cancer cells aggregate their excess centrosomes into pseudo-bipolar spindles to facilitate progression through the cell cycle [38]. The dysregulation of cell cycle progression is recognized as a fundamental mechanism driving tumorigenesis, thereby establishing cell cycle machinery regulators as viable therapeutic targets for anti-cancer interventions [39]. Wei et al. found that DNA replication of cells could be primarily affected by the ablation of KIFC1, and it has been observed that KIFC1 is not capable of translocating essential replication factors or free cytoplasmic DNA molecules into the nucleus, thereby prompting activation of the DNA damage repair mechanism and causing a prolongation of the S phase [40]. Furthermore, studies have reported the involvement of KIFC1 in promoting the initiation and progression of various carcinomas. For instance, Zhou et al. demonstrated that KIFC1 regulates the c-myc pathway to promote aerobic glycolysis in endometrial cancer [21] Similarly, Yang et al. found that KIFC1 participates in breast cancer cell proliferation by regulating glutathione metabolism [41, 42]. Li et al. revealed that KIFC1, regulated by the centrosome protein E (CENPE), actively contributes to promoting proliferation, migration, and EMT in ovarian cancer [43]. The study investigating the effects of KIFC1 inhibitors in prostate cancer also highlighted the significance of KIFC1 in cancer [25]. Moreover, there have been reports suggesting the potential of KIFC1 as a biomarker for hepatocellular carcinoma [44]. Interestingly, our cellular experiments in pancreatic cancer cells proved that after ablation of KIFC1, the apoptosis biomarkers diminished. These results confirm the conclusions of Wei et al. and keep consistent with our previous KEGG and GO enrichment analysis.

In summary, our study demonstrates that KIFC1 is frequently upregulated in various malignancies and strongly correlates with adverse patient prognoses. Additionally, KIFC1 expression levels have been shown to influence immune cell infiltration within tumor microenvironments, as well as being closely associated with tumor mutation burden and microsatellite instability in multiple cancer types. *In vitro* experiments further confirm the oncogenic role of KIFC1 in pancreatic cancer. Based on these findings, we propose that KIFC1 holds promise as a prognostic and immunotherapeutic biomarker in diverse malignancies, potentially impacting the proliferation and metastasis of pancreatic cancer cells.

## Supplementary Materials

Supplementary Figure 1
