# Supplementary material for: A comprehensive analysis of the prognostic and immunotherapeutic characteristics of KIFC1 in pan-cancer and its role in the malignant phenotype of pancreatic cancer
Source: Aging (Albany NY). 2023 Dec 18;15(24):14845–63. doi: 10.18632/aging.205311 (PMC10781448; doi:10.18632/aging.205311)
Supplement: Supplementary Figure 1 [file aging-15-205311-s001.pdf]

SUPPLEMENTARY FIGURE

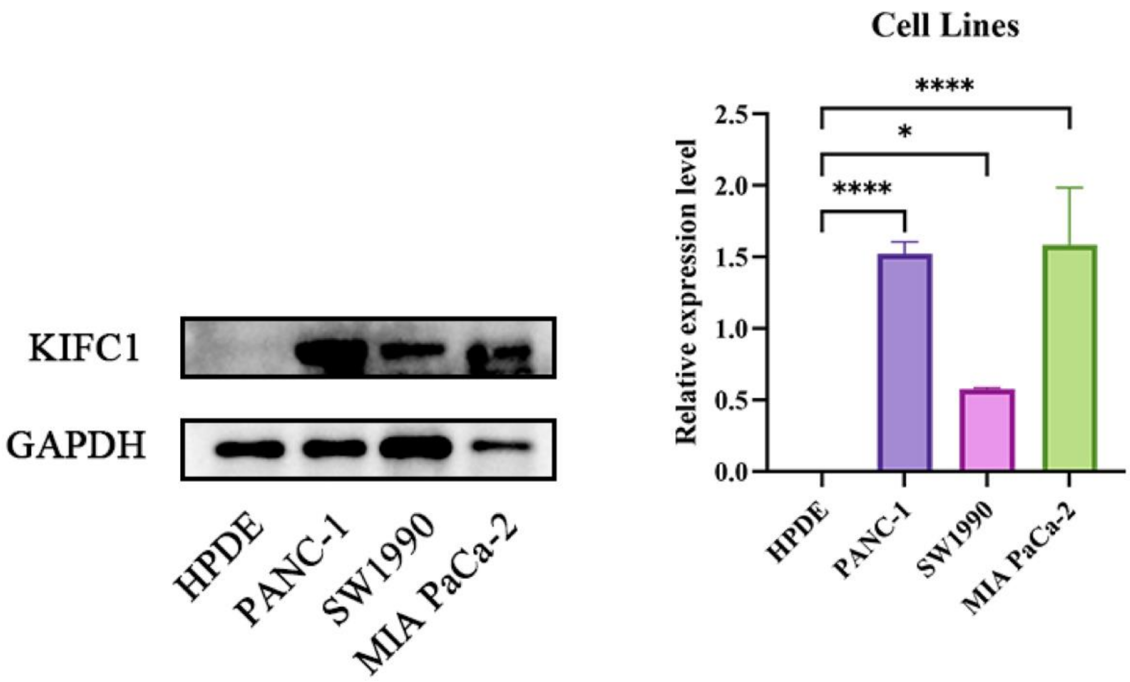

Supplementary Figure 1. The differential expression of KIFC1 in pancreatic cancer cell lines.
